# BEFORE & AFTER THE 2020 NATIONAL SHUTDOWNS: PANDEMIC EXPERIENCES OF OLDER AMERICAN ADULTS WITH & WITHOUT A DISABILITY

**DOI:** 10.1093/geroni/igac059.2992

**Published:** 2022-12-20

**Authors:** Fanni Farago

**Affiliations:** AARP, Washington D.C., District of Columbia, United States

## Abstract

Since the onset of the COVID-19 pandemic, there have been drastic changes to people’s day-to-day lives. Following the 2020 U.S. national shutdown, day-to-day life included increased social isolation, COVID-19 anxieties, disruptions to employment, and limited access to staple goods for many. Compared with the general population, the impacts of these changes are much less understood for the aging population, and particularly for older adults with a disability or chronic disease, who are at greater risk of adverse effects from COVID-19 and related circumstances. Drawing on the breaking results of the AARP Vital Voices national survey (n=2071), conducted throughout the pandemic (2019 to 2021), this poster presentation sheds light on the pandemic experiences of older American adults with and without disability or chronic disease. Interesting findings are discussed across age groups and by gender. Overall, the results show notable differences in the social connection experiences of older adults with a disability or chronic disease (n=571) pre-and-post the national shutdown, along with differences in the accessibility of household items, staple foods, and medical care in the wake of the 2020 U.S. national shutdown. The implications of these findings are discussed in the context of related literature.

